# Socioeconomic inequalities in cardiovascular mortality and the role of childhood socioeconomic conditions and adulthood risk factors: a prospective cohort study with 17-years of follow up

**DOI:** 10.1186/1471-2458-12-1045

**Published:** 2012-12-05

**Authors:** Carlijn BM Kamphuis, Gavin Turrell, Katrina Giskes, Johan P Mackenbach, Frank J van Lenthe

**Affiliations:** 1 Department of Public Health, Erasmus University Medical Centre, P.O. Box 2040, Rotterdam, CA 3000, The Netherlands; 2 School of Public Health/Institute of Health and Biomedical Innovation, Queensland University of Technology, GPO Box 2434, Brisbane, Qld, 4001, Australia

**Keywords:** Cardiovascular diseases, Socioeconomic status, Health behaviour, Life course epidemiology, Mortality determinants

## Abstract

**Background:**

The mechanisms underlying socioeconomic inequalities in mortality from cardiovascular diseases (CVD) are largely unknown. We studied the contribution of childhood socioeconomic conditions and adulthood risk factors to inequalities in CVD mortality in adulthood.

**Methods:**

The prospective GLOBE study was carried out in the Netherlands, with baseline data from 1991, and linked with the cause of death register in 2007. At baseline, participants reported on adulthood socioeconomic position (SEP) (own educational level), childhood socioeconomic conditions (occupational level of respondent’s father), and a broad range of adulthood risk factors (health behaviours, material circumstances, psychosocial factors). This present study is based on 5,395 men and 6,306 women, and the data were analysed using Cox regression models and hazard ratios (HR).

**Results:**

A low adulthood SEP was associated with increased CVD mortality for men (HR 1.84; 95% CI: 1.41-2.39) and women (HR 1.80; 95%CI: 1.04-3.10). Those with poorer childhood socioeconomic conditions were more likely to die from CVD in adulthood, but this reached statistical significance only among men with the poorest childhood socioeconomic circumstances. About half of the investigated adulthood risk factors showed significant associations with CVD mortality among both men and women, namely renting a house, experiencing financial problems, smoking, physical activity and marital status. Alcohol consumption and BMI showed a U-shaped relationship with CVD mortality among women, with the risk being significantly greater for both abstainers and heavy drinkers, and among women who were underweight or obese. Among men, being single or divorced and using sleep/anxiety drugs increased the risk of CVD mortality. In explanatory models, the largest contributor to adulthood CVD inequalities were material conditions for men (42%; 95% CI: −73 to −20) and behavioural factors for women (55%; 95% CI: -191 to −28). Simultaneous adjustment for adulthood risk factors and childhood socioeconomic conditions attenuated the HR for the lowest adulthood SEP to 1.34 (95% CI: 0.99-1.82) for men and 1.19 (95% CI: 0.65-2.15) for women.

**Conclusions:**

Adulthood material, behavioural and psychosocial factors played a major role in the explanation of adulthood SEP inequalities in CVD mortality. Childhood socioeconomic circumstances made a modest contribution, mainly via their association with adulthood risk factors. Policies and interventions to reduce health inequalities are likely to be most effective when considering the influence of socioeconomic circumstances across the entire life course and in particular, poor material conditions and unhealthy behaviours in adulthood.

## Background

Cardiovascular diseases (CVD) are some of the major causes of death in modern societies [1], and studies have shown that in almost all European countries those from a lower socioeconomic position (SEP) have higher rates of CVD mortality and morbidity [2,3]. A better understanding of the mechanisms underlying inequalities in CVD mortality is essential for devising strategies to reduce these inequalities.

Studies show that health behaviours, particularly smoking, excessive alcohol consumption, and low physical activity, contribute to the explanation of socioeconomic inequalities in CVD mortality [2,4-9]. However, for a complete understanding of socioeconomic differences in CVD mortality, a consideration of multiple factors is required [10]. Material and psychosocial factors contribute to socioeconomic inequalities in all-cause mortality, [11-13] often in conjunction with health-behaviours, and these may also be important for CVD mortality. For instance, experiencing financial problems (material factor) may lead to stress (psychosocial factor), and to cope with this, people may engage in smoking (health behaviour). To our knowledge, no study has assessed the relative contribution of material, behavioural, and psychosocial factors to inequalities in CVD mortality.

**Figure 1:**
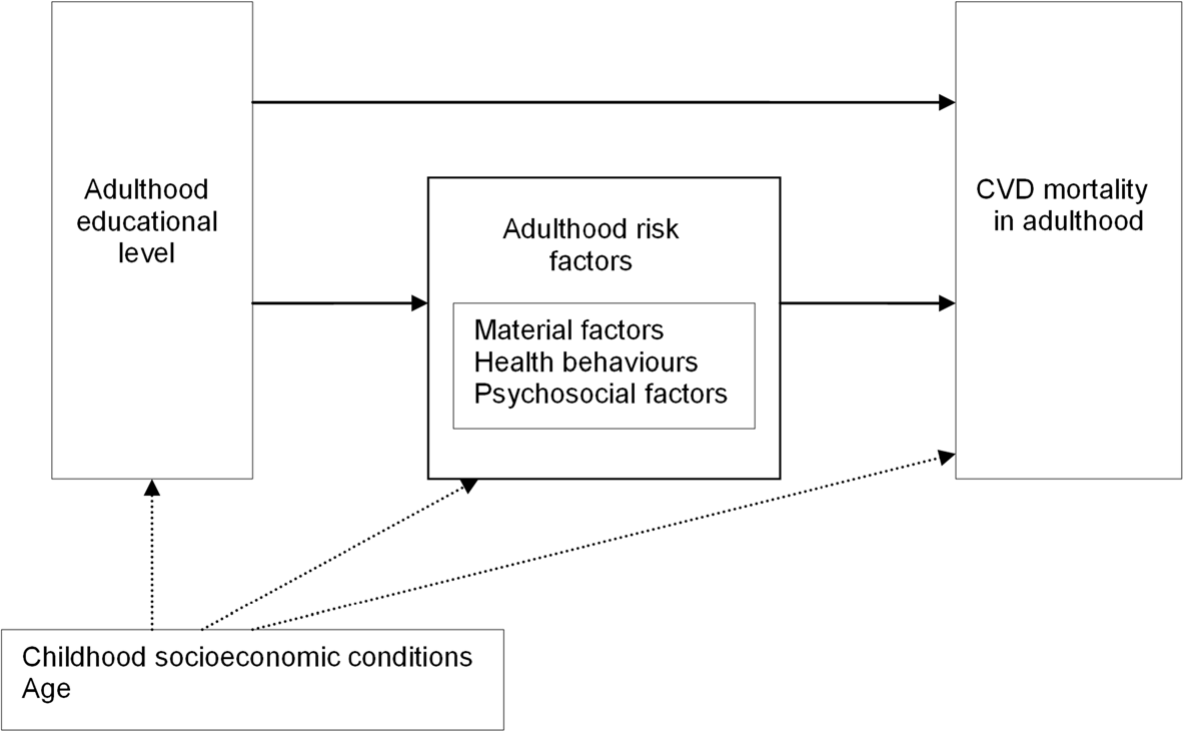
Simplified causal diagram with hypothesized associations between childhood socioeconomic conditions, adulthood educational level, adulthood risk factors and CVD mortality.

Another gap in our understanding of inequalities in CVD mortality is the role of childhood socioeconomic conditions. Adverse conditions in childhood (e.g. little parental social support, both parents being heavy smokers, illness) may occur more often in families with a low SEP, and may be associated with higher levels of risk factors in later life [14,15], thereby indirectly contributing to adulthood inequalities in CVD mortality (the so-called *pathway model*) [16]. On the other hand, it is also possible that childhood socioeconomic conditions may affect CVD risk in later life more directly, independent of adulthood risk factors. This may occur, for instance, when certain exposures, if experienced during pregnancy or infancy, lead to unfavourable and unalterable biological development, which affects CVD risk in later life (*critical period model)*[16,17]. A directed acyclic graph (DAG) was created (Figure 1) to visualise the possible associations between the factors involved in the different lifecourse models [18,19].

This present study is unique for its long follow-up time to CVD mortality, and its investigation of the role of childhood socioeconomic conditions as well as a wide range of adulthood CVD risk factors. We hypothesize that health behaviours and material and psychosocial factors each contribute to the explanation of adulthood socioeconomic inequalities in CVD mortality. Furthermore, we hypothesize that childhood socioeconomic conditions will contribute to the explanation of adulthood socioeconomic inequalities in CVD mortality, partly indirectly via their association with risk factors in later life, and partly directly, independent of adulthood risk factors.

## Methods

Longitudinal data were used from the GLOBE study conducted in the Netherlands (these data are available from the first author upon request). Detailed information about the study design and sampling methods are provided elsewhere [20,21]. In 1991, a random sample of 27,027 non-institutionalised Dutch persons aged 15–75 years living in the city of Eindhoven and its surrounding area was drawn from the municipal population register. This sample was sent a postal questionnaire (response rate 70.1%, N=18,793). All measures described below were self-reported in the baseline questionnaire.

### Adulthood and childhood socioeconomic position

In this study, all ‘adulthood’ measures (i.e. adulthood socioeconomic position as well as adulthood risk factors) pertain to people age 40 years or older (since the analyses were restricted to respondents in this age range at baseline, see below), and ‘childhood’ refers to the age of 12 years. *Adulthood socioeconomic position (SEP)* was determined by the respondents’ highest attained education level, with four categories: 1-low (primary education), 2- (lower professional and intermediate general education), 3- (intermediate professional and higher general education), 4-high (higher professional education and university) [22].

Participants were asked to retrospectively recall the occupational title of their father when they were twelve years of age, or, if their father was unemployed, the title of his last occupation (*childhood socioeconomic conditions*). These data were classified according to the Erikson, Goldthorpe, and Portocarero scheme, [23] and three categories were created: professionals (top-level management, advanced academic competencies, high level of independence), white-collar (middle management, routine non-manual work), and blue-collar occupations (skilled and unskilled manual work).

### Health-behaviours

*Smoking status* was categorised as never, former and current smoker based on the response to the question “Do you smoke?” [12,13].

*Physical activity* was based on three questions, asking for time spent per week (never, <1 hour, 1–2 hour, 2≥ hour; analysed as 0 hour, 0.5 hour, 1.5 hour, and 2.5 hour) on transport-related activity (walking, cycling), leisure time physical activity (gardening, walking, cycling), and sports activity [13]. Time spent on transport and leisure activity was summed as ‘moderate physical activity’. Participants were classified as inactive (no sports and 0–1 hours of moderate physical activity), little active (either no sports and 1–2 hours of moderate physical activity, or <1 hours of sports and 0–1 hours of moderate physical activity), moderately active (2.5-3.5 hours of moderate physical activity and sports combined), or active (at least 3,5 hours of sports or moderate physical activity combined) [8].

*Alcohol consumption* was calculated from two questions, one asking for the number of days per week drinking any alcoholic drinks, and the second asking for the number of alcohol drinks (units) consumed on such a day [12,13]. Participants were categorised as abstainers (0 units/week), light drinkers (1–7 units for women, 1–10 units for men), moderate drinkers (8–14 units for women, 11–21 units for men), and heavy drinkers (>14 units for women, >21 units for men) [8,24].

*Body mass index (BMI)* was calculated from self-reported weight in kilograms/self-reported height in meters^2^, and respondents were classified as being underweight (BMI ≤20), average weight (BMI 20–25), overweight (BMI 25–30), or obese (BMI 30≥) [25].

### Material circumstances

Four items that are indicators of the financial situation of the household were measured and have been applied in several studies among the GLOBE cohort: [12,13]*type of health insurance* (private, public), *car ownership* (yes, no), *housing tenure* (rented house, house owner), and *financial problems* with paying bills for food, rent, electricity etc. over the preceding year (no, some, big problems) [12,13]. *Adverse neighbourhood conditions* were measured by four questions about noise from neighbours, noise from traffic, smells, and vandalism in the neighbourhood (no, 1≥ adverse conditions) [12,13]. *Adverse housing conditions* were measured by three questions on cold, mould, and dampness in the house (no, 1≥ adverse conditions) [12,13].

### Psychosocial factors

Data describing psychosocial factors included indicators of *marital status* (married, single, divorced, widowed) and *negative life events*. Respondents were asked if they experienced each of nine *negative life events* in the preceding year, such as a decline in financial position, severe disease of partner, and divorce (no event, 1 event, 2≥ events) [13,26]. Furthermore, *use of medicine for anxiety* (yes, no) and whether respondents had experienced *depression, severe nervousness or burn-out* over the last five years (yes, no) were applied as psychosocial indicators [11].

### CVD mortality and data linkage

Cause-specific mortality data were obtained from Statistics Netherlands. Causes of death were coded in accordance with the 9th and 10th version of the International Classification of Diseases (ICD), with codes 390–459 (ICD 9) or I00-I99 (ICD 10) for cardiovascular diseases. For each GLOBE respondent, the mortality follow-up extended from the baseline survey (April 1st, 1991) until October 15, 2007. If respondents died in the follow-up period, their death date was used to calculate survival time. If they did not die, survival time was calculated with October 15, 2007 as the final date. If respondents moved out of the Netherlands between baseline and October 15, 2007, survival time was calculated from the baseline until the date they emigrated.

### Analytic sample

Baseline respondents were excluded from the current analyses if they were 1) younger than 40 years of age at baseline (2 946 men, 2 902 women), 2) reported in the baseline questionnaire that they had experienced severe heart problems or a heart attack (599 men, 314 women) or a stroke (100 men, 48 women) in the preceding five years, or 3) had a missing value for adulthood SEP (199 men and 234 women) (these categories partly overlapped). The current analyses were based on the remaining 11 701 participants (5 395 men and 6 306 women).

### Statistical analyses

Analyses were performed in SPSS [27], the significance level used was .05, and all analyses were adjusted for age. Analyses were undertaken for men and women separately, since the socioeconomic distribution of risk factors and their relative importance for explaining CVD inequalities may differ for men and women [28]. For each of the risk factors and for childhood socioeconomic conditions, respondents with a missing value remained in the analyses as a separate category (see Additional file 1 for prevalence rates of missing values).

Using Cox proportional hazard models, we assessed associations between adulthood SEP and CVD mortality. We assessed the distribution of childhood socioeconomic conditions and adulthood risk factors by adulthood SEP using Chi-square tests, and associations of childhood socioeconomic conditions with CVD mortality (adjusted for age), and adulthood risk factors with CVD mortality (adjusted for age, childhood socioeconomic conditions and adulthood SEP) by Cox proportional hazard models.

Factors that were significantly related to cardiovascular mortality and that varied by adulthood SEP were included in the following models: 1) adulthood SEP; 2) adulthood SEP + childhood socioeconomic conditions; 3) adulthood SEP + material factors; 4) adulthood SEP + behavioural factors; 5) adulthood SEP + psychosocial factors; 6) adulthood SEP + all adulthood risk factors; 7) adulthood SEP + childhood socioeconomic conditions + all adulthood risk factors. For each model, the percent change in relative hazards for SEP-groups compared to model 1 was evaluated. A 95% CI was calculated around the percentage attenuation using a bias-corrected accelerated bootstrap method with 1000 re-samplings in the statistical program R [29].

## Results

A total of 542 men (9.7%) and 403 women (6.2%) died from CVD during the 17-year follow-up. A low adulthood SEP was associated with an increased risk of CVD mortality among both men (HR 1.84, 95% CI 1.41-2.39) and women (HR 1.80, 95% CI 1.04-3.10).

### Childhood socioeconomic conditions and adulthood risk factors by adulthood SEP

Adulthood SEP was significantly associated with childhood socioeconomic conditions and with all adulthood material, psychosocial and behavioural factors among both men and women (see Additional file 1). Associations were mostly in expected directions, i.e. poorer childhood socioeconomic conditions, less favourable material circumstances (e.g. rent a house, no car, financial problems, problems with physical housing conditions) and more unhealthy behaviours (e.g. being physically inactive, overweight or obese, smoking) among the low SEP groups. In contrast, among women, two adverse psychosocial factors (i.e. having experienced 2≥ negative life events, and depression/nervousness) were more prevalent among those with a high adulthood SEP, but among men, adverse psychosocial factors were more prevalent among those with a low adulthood SEP.

### Childhood socioeconomic conditions, adulthood risk factors and CVD mortality

As presented in Table 1, those with poorer childhood socioeconomic conditions were more likely to die from CVD in adulthood, but this only reached statistical significance among men from the poorest childhood socioeconomic circumstances. About half of the investigated adulthood risk factors showed significant associations with CVD mortality among both men and women, namely renting a house, experiencing financial problems, smoking, physical activity and marital status. Alcohol consumption showed a U-shaped relationship with CVD mortality among women, with an increased risk for both abstainers and heavy drinkers. Also, BMI showed a U-shaped relationship with CVD mortality among women: those who were underweight or obese were at higher risk for CVD mortality. BMI was not significant among men, although –in contrast to women- underweight seemed to have a protective effect against CVD mortality. Further, among men, being single or divorced, and using sleep/anxiety drugs increased the risk of CVD mortality, whereas among women, those who were widowed or had a history with depression/nervousness were at an increased risk. No significant associations with CVD mortality were found for type of health insurance, problems with neighbourhood and housing conditions, and negative life events (among men and women), alcohol consumption, BMI and having experienced depression/nervousness (among men), and use of sleep/anxiety drugs (among women).

**Table 1:** **Hazard ratios (HR’s) for cardiovascular diseases (CVD) mortality by childhood socioeconomic conditions**^**a **^**(adjusted for age)**^**b**^**, and HR’s for CVD mortality by adulthood risk factors (adjusted for age, childhood socioeconomic conditions and adulthood SEP**^**c**^**)**^**b**^**, for men and women**

	**Men (n=5395)**		**Women (n=6306)**	
	**HR (95% CI) for CVD mortality**	**p**	**HR (95% CI) for CVD mortality**	**p**
*Childhood socioeconomic conditions*^b^				
Occupation of respondent’s father (professional = 1.00)		**.021**		**.010**
white collar	1.09 (0.78-1.51)		1.00 (0.66-1.44)	
blue collar	1.34 (1.01-1.77)		1.10 (0.78-1.53)	
*Material conditions*				
House renter (home owner = 1.00)	1.31 (1.08-1.59)	**.016**	1.35 (1.07-1.69)	**.022**
No car (car = 1.00)	1.37 (1.10-1.72)	**.018**	1.27 (1.01-1.59)	.100
Public health insurance (private = 1.00)	1.19 (0.97-1.48)	.194	1.17 (0.93-1.47)	.382
Financial problems^a^ (no = 1.00)				
Some financial problems	1.08 (0.86-1.37)	**.023**	1.31 (1.02-1.68)	**.013**
Many financial problems	1.74 (1.11-2.72)		1.82 (1.18-2.80)	
Problems with neighbourhood conditions (no=1.00)	0.94 (0.78-1.14)	.816	1.02 (0.81-1.28)	.980
Problems with housing conditions (no=1.00)	1.02 (0.82-1.28)	.979	1.13 (0.88-1.44)	.592
*Health-behaviours*				
Smoking (never = 1.00)				
former	0.97 (0.69-1.36)	**.000**	0.80 (0.61-1.06)	**.000**
current	1.85 (1.33-2.57)		1.87 (1.48-2.37)	
Physical activity (active = 1.00)				
moderately active	1.16 (0.94-1.42)	**.001**	1.31 (1.01-1.70)	**.000**
little active	1.52 (1.17-1.97)		1.40 (1.03-1.90)	
inactive	1.71 (1.30-2.26)		2.09 (1.57-2.79)	
Alcohol consumption (light = 1.00)				
abstainer	1.12 (0.88-1.44)	.247	1.80 (1.39-2.34)	**.000**
moderate drinker	0.98 (0.78-1.24)		1.22 (0.81-1.84)	
heavy drinker	1.36 (1.02-1.82)		2.01 (1.27-3.18)	
BMI (normal (20-25) =1.00)				
underweight (<20)	0.47 (0.21-1.05)	.063	1.93 (1.28-2.91)	**.000**
overweight (25.01-30)	1.09 (0.91-1.32)		0.92 (0.72-1.17)	
obese (>30)	1.39 (0.94-2.07)		1.66 (1.22-2.27)	
*Psychosocial factors*				
Negative life events (no = 1.00)				
1> negative life events	1.10 (0.91-1.34)	.743	0.98 (0.77-1.25)	.234
2> negative life events	0.98 (0.76-1.26)		1.30 (0.99-1.72)	
Marital status (married =1.00)				
single	1.51 (1.03-2.21)	**.050**	1.31 (0.88-1.95)	**.002**
divorced	1.45 (1.02-2.07)		1.46 (0.95-2.26)	
widowed	1.20 (0.82-1.75)		1.51 (1.18-1.93)	
Use of sleep/anxiety drugs (no= 1.00)	1.51(1.10-2.08)	.**038**	0.98 (0.72-1.33)	.984
Depression, nervousness (no=1.00)	1.20 (0.91-1.60)	.427	1.34 (1.00-1.78)	.090

SEP, socioeconomic position; CVD, cardiovascular diseases; HR, hazard ratio; CI, confidence interval; BMI, body mass index (kg/m^2^).

^a ^Childhood socioeconomic conditions were determined by the occupation of the respondent’s father when the respondent was 12 years of age, with 1=professional, 2=white collar, 3=blue collar.

^b ^As shown in Figure 1, age, adulthood SEP, and childhood SEP are confounders in the associations of adulthood risk factors with CVD mortality. For the association of childhood socioeconomic conditions with CVD mortality, age is the only confounder.

^c ^Adulthood socioeconomic position was determined by the respondent’s highest attained educational level, with 1= primary, 2= lower secondary, 3= higher secondary, 4=tertiary.

^d ^For childhood socioeconomic conditions as well as all adulthood risk factors, missing values were retained in the analyses as a separate category. For childhood socioeconomic conditions, we reported the HR for the category of missing values in the table, since the proportion of missing values was high, i.e. 12.1% for men and 13.8% for women. For adulthood risk factors, the proportion of missing values was generally low (see Additional file 1), i.e. ranging from 0.1% for physical activity to 5.7% for alcohol consumption among men, and from 0.5% for physical activity to 7.8% for alcohol consumption among women.

### Models for explaining adulthood SEP inequalities in CVD mortality

Explanatory models in Table 2 (men) and Table 3 (women) show to what extent associations between adulthood SEP and CVD mortality were explained by childhood socioeconomic conditions and adulthood risk factors. The highest risk for CVD mortality as observed among those with the lowest adulthood SEP (model 1) reduced insignificantly by 15% (CI: -40% to 5%) for men and 11% (CI: -74% to 31%) for women when childhood socioeconomic conditions were taken into account (model 2). Of the three groups of adulthood risk factors, material conditions made the largest contribution to the explanation of adulthood SEP inequalities in CVD mortality among men (42%; CI: -73% to −20%), and behavioural factors among women (55%; CI: -191% to −30%). When material, behavioural and psychosocial factors were all included (model 6), the HR for the lowest adulthood SEP reduced by 52% (CI: -94% to −33%) among men, and by 73% (CI: -230% to −34%) among women. This model was further adjusted for childhood socioeconomic conditions (model 7), which led to a total reduction of the HR among those with the lowest adulthood SEP of 60% (CI: -109% to −32%) among men and 76% (CI: -232% to −29%) among women.

**Table 2:** **Role of material, psychosocial and behavioural factors and childhood socioeconomic conditions**^**a **^**in explaining associations of adulthood SEP**^**b **^**with CVD mortality (adjusted for age), among men (n=5395)**

	**Adulthood SEP**						
	**1- low (n=1270)**		**2- middle low (n=1795)**		**3- middle high (n=1088)**		**4- high (n=1242)**
**Explanatory models**^**c**^	**HR (95% CI)**	**%attentuation**^**d**^**(95% CI)**	**HR (95% CI)**	**%attentuation**^**d**^**(95% CI)**	**HR (95% CI)**	**%attentuation**^**d**^**(95% CI)**	**HR (95% CI)**
Died from CVD (n (%))	184 (14.5)		156 (8.7)		97 (8.9)		80 (6.4)
Model 1:	1.84 (1.41-2.39)		1.32 (1.01-1.73)		1.31 (0.97-1.76)		1.00
adulthood SEP							
Model 2:	1.71 (1.29-2.26)	−15% (−40 to 5)	1.26 (0.95-1.66)	−19% (−120 to 14)	1.27 (0.94-1.71)	−13% (−112 to 29)	1.00
adulthood SEP							
+ childhood conditions							
Model 3:	1.49 (1.12-1.98)	−42% (−73 to −20)	1.16 (0.88-1.54)	−50% (−252 to −7)	1.22 (0.91-1.65)	−29% (−259 to 87)	1.00
adulthood SEP							
+ material factors^e^							
Model 4:	1.70 (1.30-2.21)	−17% (−39 to −12)	1.28 (0.98-1.68)	−13% (−92 to 9)	1.33 (0.99-1.79)	-	1.00
adulthood SEP							
+behavioural factors^f^							
Model 5:	1.76 (1.34-2.29)	−10% (−21 to −1)	1.32 (1.00-1.72)	-	1.32 (0.99-1.78)	-	1.00
adulthood SEP							
+psychosocial factors^g^							
Model 6:	1.40 (1.05-1.86)	−52% (−94 to −33)	1.17 (0.88-1.54)	−47% (−249 to −1)	1.26 (0.94-1.71)	−16% (−137 to 46)	1.00
adulthood SEP							
+ material + behavioural							
+ psychosocial factors							
Model 7:	1.34 (0.99-1.82)	−60% (−109 to −32)	1.13 (0.85-1.51)	−59% (−291 to −2)	1.24 (0.92-1.68)	−23% (−202 to 64)	1.00
adulthood SEP							
+ childhood conditions							
+ material + behavioural							
+ psychosocial factors							
Direct contribution of childhood conditions (i.e. independent of adulthood risk factors)		8% (60–52)		12% (59–47)		7% (23–16)	
Direct contribution of adulthood risk factors (i.e. independent of childhood conditions)		45% (60–15)		40% (59–19)		10% (23–13)	
Indirect contribution of childhood conditions, i.e. via adulthood risk factors		7% (15+52-60)		7% (19+47-59)		6% (13+16-23)	

SEP, socioeconomic position; CVD, cardiovascular diseases; HR, hazard ratio; CI, confidence interval; BMI, body mass index (kg/m^2^).

^a ^Childhood socioeconomic conditions were determined by the occupation of the respondent’s father when the respondent was 12 years of age, with 1=professional, 2=white collar, 3=blue collar.

^b ^Adulthood socioeconomic position was determined by the respondent’s highest attained educational level, with 1= primary, 2= lower secondary, 3= higher secondary, 4=tertiary.

^c ^Only factors that were significantly associated with CVD mortality and unequally distributed across adulthood SEP groups were included in the explanatory models, and all models were adjusted for age.

^d ^ The percentages show the reduction in harzard ratio (HR) compared to model 1, per SEP group. For instance, the reduction in the OR for the lowest adulthood SEP group when adding childhood socioeconomic conditions to the first model, is [(1.84-1.71)/(1.84-1.00)]*100 = 15%.

^e ^Material factors: car ownership, housing tenure, and financial problems.

^f ^Behavioural factors: smoking, physical activity.

^g ^Psychosocial factors: marital status, use of sleep/anxiety drugs.

**Table 3:** **Role of material, psychosocial and behavioural factors and childhood socioeconomic conditions**^**a **^**in explaining associations of adulthood SEP**^**b **^**with CVD mortality (adjusted for age), among women (n=6306)**

	**Adulthood SEP**						
	**1- low (n=1961)**		**2- middle low (n=3079**		**3- middle high (n=774)**		**4-high (n=492)**
**Explanatory models**^**c**^	**HR (95% CI)**	**%attentuation**^**d**^ **(95% CI)**	**HR (95% CI)**	**%attentuation**^**d**^ **(95% CI)**	**HR (95% CI)**	**%attentuation**^**d**^ **(95% CI)**	**HR (95% CI)**
Died from CVD (n (%))	187 (9.5)		140 (4.5)		38 (4.9)		14 (2.8)
Model 1:	1.80 (1.04-3.10)		1.39 (0.80-2.41)		1.42 (0.77-2.62)		1.00
adulthood SEP							
Model 2:	1.71 (0.97-3.01)	−11% (−74 to 31)	1.37 (0.78-2.41)	−5% (−132 to 102)	1.42 (0.77-2.64)	-	1.00
adulthood SEP							
+ childhood conditions							
Model 3:	1.50 (0.86-2.62)	−38% (−135 to −13)	1.24 (0.71-2.16)	−38% (−284 to 233)	1.34 (0.73-2.49)	−19% (−128 to 116)	1.00
adulthood SEP							
+ material factors^e^							
Model 4:	1.36 (0.78-2.38)	−55% (−191 to −28)	1.20 (0.69-2.09)	−49% (−414 to 244)	1.35 (0.73-2.50)	−17% (−167 to 113)	1.00
adulthood SEP							
+behavioural factors^f^							
Model 5:	1.75 (1.00-3.04)	−6% (−38 to 21)	1.38 (0.79-2.41)	−3% (−74 to 121)	1.40 (0.76-2.59)	−5% (−65 to 55)	1.00
adulthood SEP							
+psychosocial factors^g^							
Model 6:	1.22 (0.69-2.18)	−73% (−230 to −34)	1.13 (0.64-1.99)	−67% (−619 to 235)	1.29 (0.70-2.40)	−31% (−230 to 139)	1.00
adulthood SEP							
+ material + behavioural							
+ psychosocial factors							
Model 7:	1.19 (0.66-2.15)	−76% (−232 to −29)	1.12 (0.63-2.02)	−69% (−446 to 468)	1.29 (0.71-2.47)	−31% (−230 to 294)	1.00
adulthood SEP							
+ childhood conditions							
+ material + behavioural							
+ psychosocial factors							
Direct contribution of childhood conditions (i.e. independent of adulthood risk factors)		3% (76–73)		2% (69–67)		0% (31–31)	
Direct contribution of adulthood risk factors (i.e. independent of childhood conditions)		65% (76–11)		64% (69–5)		31% (24–0)	
Indirect contribution of childhood conditions, i.e. via adulthood risk factors		8% (11+73-76)		3% (5+67-69)		0% (0+31-31)	

SEP, socioeconomic position; CVD, cardiovascular diseases; HR, hazard ratio; CI, confidence interval; BMI, body mass index (kg/m^2^).

^a ^Childhood socioeconomic conditions was determined by the occupation of the respondent’s father when the respondent was 12 years of age, with 1=professional, 2=white collar, 3=blue collar.

^b ^ Adulthood socioeconomic position was determined by the respondent’s highest attained educational level, with 1= primary, 2= lower secondary, 3= higher secondary, 4=tertiary.

^c ^Only factors that were significantly associated with CVD mortality and unequally distributed across adulthood SEP groups were included in the explanatory models. All models were adjusted for age.

^d ^The percentages show the reduction in harzard ratio (HR) compared to model 1, per SEP group. For instance, the reduction in the OR for the lowest adulthood SEP group when adding childhood SEP to the first model, is [(1.80-1.71)/(1.80-1.00)] * 100 = 11%.

^e ^Material factors: housing tenure, and financial problems.

^f ^Behavioural factors: smoking, physical activity, alcohol consumption, BMI.

^g ^Psychosocial factors: marital status.

In sum, the total explained effect of adulthood SEP on CVD mortality (60% for men and 76% for women) was largely accounted for by risk factors in adulthood. Adulthood risk factors explained the greater part of the inequalities in CVD mortality, independent of childhood socioeconomic conditions (45% for men and 65% for women). Childhood socioeconomic conditions made a modest contribution to the explanation of inequalities in CVD mortality, i.e. mainly via their association with adulthood risk factors (7% for men and 8% for women). For men 8%, and for women 3%, of the total explained effect of adulthood SEP on CVD mortality was due to a direct effect of childhood socioeconomic circumstances on CVD mortality (i.e. independent of adulthood risk factors). Childhood socioeconomic conditions were most important for explaining the increased risk of dying from CVD among men with the second lowest adulthood SEP (12% directly, and 7% via adulthood risk factors).

## Discussion

The purpose of this study was to gain a better insight in the role of childhood socioeconomic conditions as well as a wide range of adulthood risk factors for explaining CVD inequalities in adulthood. We showed that inequalities in CVD mortality were largely explained by adulthood material and behavioural risk factors, and less by psychosocial factors. Childhood socioeconomic circumstances made a modest contribution to the explanation of adulthood CVD inequalities, mainly via their association with adulthood risk factors.

Our conclusion that adulthood risk factors play a central role in the explanation of adulthood SEP inequalities in CVD mortality is consistent with previous work [5,8,30]. We did not find critically different results for men and women, however, material conditions were most important for explaining adulthood SEP inequalities in CVD mortality among men, whereas behavioural factors were most important among women. Results indicated that the explanatory power of adulthood risk factors was partly due to the association of childhood socioeconomic conditions with these risk factors [10]. This finding is in line with the pathway model and not with the critical period model, as poor childhood socioeconomic circumstances were associated with an increased CVD risk in later life *via* –and hardly independent of- adulthood risk factors [16].

Highest attained educational level was used as the indicator of adulthood SEP. Level of education is considered a good indicator of SEP in the Netherlands, and therefore often applied [23]. Arguably, of all possible adulthood SEP indicators, education is most closely related in time to one’s milieu of origin. Due to this, we may have underestimated the role of childhood socioeconomic conditions in the models where we adjusted for adulthood SEP. On the other hand, the correlation between the two SEP indicators was only 0.345 in our study, which shows that both indicators are to a large extent measuring different underlying concepts.

In additional analyses, we assessed the relative importance of the four health-behaviours to the explanation of adulthood SEP inequalities in CVD mortality, and whether this differed for men and women [see Additional file 2. Among men, smoking was the most important health behaviour for CVD inequalities, as it explained 17% of the gradient according to adulthood SEP. Among women, smoking explained a similar proportion of the gradient (18%), but physical activity (25%) and alcohol consumption (25%) made larger contributions. The few other studies that reported on the relative importance of behavioural risk factors for CVD mortality for men and women separately found similar results with regard to smoking, in that smoking was relatively more important for men than women [5,9]. Differences between men and women regarding the importance of other health-behaviours were less consistent. Strand and colleagues (2004) also showed that BMI contributed more to the inequalities among women, but they did not observe a gender difference in relative importance of physical activity (alcohol was not measured) [9]. However, overall, studies concur that health-behaviours like smoking, alcohol consumption, and physical activity contribute to inequalities in CVD mortality to a large extent, but which type of health-behaviour is most important differs by country, specific population, time of the survey etc.[5,8,9,30,31].

Since the absolute prevalence of risk factors affect the attributable mortality, we compared the prevalence rates in our study sample to those in the general population of the Netherlands, showing that these rates were of similar magnitude. In our analytic sample (all 40 years or older, data collected in 1991), rates of current smokers were 41.7% (men) and 28.0% (women), compared to 42,8% (men) and 31,5% (women) in the general population (of 12 years and older, data collected in 1990) (http://statline.cbs.nl). Further, rates of overweight/obesity were 46.0% (men) and 38.9% (women) in our sample, compared to 39,6% (men) and 30,6% (women) in the general population (of 20 years of age and older). Rates of overweight/obesity were somewhat higher in our sample, but this could be due to the relatively older age (40> years) compared to the age of the sample of the general population (20> years).

### Strengths and limitations

To our knowledge, this is the first study to simultaneously investigate the contribution of adulthood health behaviours, material conditions, and psychosocial factors, and childhood socioeconomic conditions, to adulthood SEP inequalities in CVD mortality. The measurement of a large number of risk factors is a strength, but may also be a limitation, since, due to space limitations in the postal survey, every risk factor could only be measured with a limited number of items. Furthermore, the independent variables examined were only measured once, and were based on self-reports, which may have resulted in biased responses. Psychosocial conditions could have had more effect on the explanation of socioeconomic inequalities in CVD mortality when measured more specifically (e.g. by locus of control, number of good friends, social participation, social networks), although the psychosocial factors we included (i.e. indicators for marital status, anxiety and depression) have been used previously [11]. Father’s occupation at the respondent’s age of twelve was measured retrospectively, which may have caused underestimated associations compared to prospective measures of childhood SEP. However, retrospective reports of childhood circumstances have shown to be relatively reliable [32]**.** In the baseline questionnaire, participants were asked whether they experienced severe heart problems, a heart attack or stroke over the last five years. We excluded these participants from the analysis as they may have changed to healthier behaviours post-baseline while still having a higher chance of dying from CVD. However, we had no information about earlier histories of CVD, hence the analyses may still have included participants who experienced heart problems or a stroke and later changed to a healthier lifestyle, which might have underestimated the contribution of behavioural factors.

## Conclusions and implications

Adulthood risk factors played a major role in the explanation of socioeconomic inequalities in CVD mortality. Childhood socioeconomic circumstances made a modest contribution, mainly via their association with adulthood risk factors. More research is needed to better understand to what extent and how socioeconomic adversity in childhood may have long-lasting negative influences on adulthood risk factors and health, since this might indicate whether primary prevention of behavioural risk factors through interventions in youth is effective for CVD prevention. Policies and interventions to reduce health inequalities are likely to be most effective when considering the influence of socioeconomic circumstances across the entire life course, and in particular, poor material conditions and unhealthy behaviours in adulthood.

## Competing interests

The authors' declare that they have no competing interests.

## Authors' contribution

FJvL, CBMK, and JPM conceived the study. CBMK and GT performed the analyses. CBMK wrote the manuscript. All authors critically reviewed the manuscript for important intellectual content, and approved the final version of the manuscript.

## Pre-publication history

The pre-publication history for this paper can be accessed here:

http://www.biomedcentral.com/1471-2458/12/1045/prepub

## Supplementary Material

Additional file 1 **This Table shows the prevalence of childhood socioeconomic conditions, and adulthood material, psychosocial and behavioural risk factors by adulthood SEP**^**a**^**, for men and women.** Click here for file

Additional file 2 This Table shows the relative importance of the four health-behaviours to the explanation of adulthood SEP inequalities in CVD mortality, for men and women. Click here for file
