# Supplementary material for: Socioeconomic inequalities in cardiovascular mortality and the role of childhood socioeconomic conditions and adulthood risk factors: a prospective cohort study with 17-years of follow up
Source: BMC Public Health. 2012 Dec 5;12:1045. doi: 10.1186/1471-2458-12-1045 (PMC3539932; doi:10.1186/1471-2458-12-1045)
Supplement: Additional file 1 — This Table shows the prevalence of childhood socioeconomic conditions, and adulthood material, psychosocial and behavioural risk factors by adulthood SEPa, for men and women. [file 1471-2458-12-1045-S1.doc]

**Additional file 1**

**Supplementary Table A.** Childhood socioeconomic conditions, and adulthood material, psychosocial and behavioural risk factors by adulthood SEP^a^, for men and women.

|  | **Adulthood SEP**^b^ **(men)** | | | | |  | **Adulthood SEP**^b^ **(women)** | | | | |  |
| --- | --- | --- | --- | --- | --- | --- | --- | --- | --- | --- | --- | --- |
|  | **TOTAL MEN** | **1 – low** | **2** | **3** | **4- high** | **p^b^** | **TOTAL**  **WOMEN** | **1 – low** | **2** | **3** | **4- high** | **p^b^** |
|  | (n=5395) | (n=1270) | (n=1795) | (n=1088) | (n=1242) |  | (n=6306) | (n=1961) | (n=3079) | (n=774) | (n=492) |  |
|  |  | % | % | % | % |  |  | % | % | % | % |  |
| *Childhood socioeconomic conditions* |  |  |  |  |  |  |  |  |  |  |  |  |
| Occupation of respondent’s father |  |  |  |  |  | <.0001 |  |  |  |  |  | <.0001 |
| professional | 14.5 | 2.6 | 9.6 | 18.7 | 30.2 |  | 13.1 | 4.6 | 10.6 | 26.0 | 42.1 |  |
| white collar | 19.0 | 10.3 | 17.8 | 22.4 | 26.5 |  | 18.0 | 10.4 | 19.6 | 27.0 | 24.4 |  |
| blue collar | 54.5 | 63.9 | 61.4 | 51.8 | 37.0 |  | 55.1 | 62.5 | 58.3 | 40.3 | 29.1 |  |
| missing | 12.1 | 23.1 | 11.2 | 7.1 | 6.4 |  | 13.8 | 22.6 | 11.5 | 6.7 | 4.5 |  |
|  |  |  |  |  |  |  |  |  |  |  |  |  |
| *Material conditions* |  |  |  |  |  |  |  |  |  |  |  |  |
| Housing tenure |  |  |  |  |  | <.0001 |  |  |  |  |  | <.0001 |
| Renter | 43.6 | 66.7 | 50.1 | 35.9 | 17.3 |  | 46.8 | 64.2 | 44.5 | 28.9 | 19.3 |  |
| Home owner | 54.6 | 29.2 | 48.6 | 62.5 | 82.3 |  | 50.7 | 31.1 | 53.8 | 69.0 | 80.7 |  |
| missing | 1.8 | 4.1 | 1.3 | 1.6 | 0.4 |  | 2.6 | 4.7 | 1.7 | 2.1 | 0.0 |  |
| Car ownership |  |  |  |  |  | <.0001 |  |  |  |  |  | <.0001 |
| No car | 12.1 | 24.6 | 11.4 | 7.6 | 4.4 |  | 18.3 | 30.0 | 14.3 | 10.9 | 7.9 |  |
| Car | 86.5 | 72.2 | 87.6 | 91.3 | 95.4 |  | 79.3 | 65.2 | 83.9 | 88.5 | 91.9 |  |
| missing | 1.4 | 3.2 | 1.1 | 1.1 | 0.2 |  | 2.5 | 4.8 | 1.8 | 0.6 | 0.2 |  |
| Type of health insurance |  |  |  |  |  | <.0001 |  |  |  |  |  | <.0001 |
| Public | 50.3 | 86.1 | 63.5 | 35.1 | 8.1 |  | 60.6 | 77.8 | 60.9 | 40.6 | 21.5 |  |
| Private | 49.5 | 13.5 | 36.4 | 64.6 | 91.8 |  | 39.0 | 21.4 | 38.9 | 59.0 | 77.8 |  |
| missing | 0.2 | 0.5 | 0.1 | 0.3 | 0.2 |  | 0.4 | 0.8 | 0.2 | 0.4 | 0.6 |  |
| Financial problems |  |  |  |  |  | <.0001 |  |  |  |  |  | <.0001 |
| No problems | 78.2 | 64.9 | 75.3 | 82.8 | 92.2 |  | 74.8 | 64.4 | 77.3 | 82.2 | 88.6 |  |
| Some financial problems | 17.5 | 25.4 | 21.4 | 14.6 | 6.4 |  | 19.1 | 26.0 | 17.7 | 13.4 | 8.9 |  |
| Big financial problems | 3.0 | 6.7 | 2.5 | 1.9 | 1.0 |  | 4.1 | 6.3 | 3.5 | 2.6 | 1.6 |  |
| missing | 1.2 | 3.0 | 0.8 | 0.6 | 0.3 |  | 2.1 | 3.3 | 1.5 | 1.8 | 0.8 |  |
| Neighbourhood problems |  |  |  |  |  | <.0001 |  |  |  |  |  | <.0001 |
| yes | 32.7 | 30.3 | 32.9 | 36.9 | 31.0 |  | 31.4 | 28.5 | 30.8 | 35.3 | 40.4 |  |
| no | 65.3 | 65.0 | 65.2 | 62.1 | 68.6 |  | 65.1 | 65.4 | 66.4 | 62.7 | 59.3 |  |
| missing | 2.0 | 4.6 | 1.9 | 1.0 | 0.4 |  | 3.5 | 6.1 | 2.8 | 2.1 | 0.2 |  |
| Problems with housing conditions |  |  |  |  |  | <.0001 |  |  |  |  |  | <.0001 |
| Yes | 19.5 | 25.7 | 20.9 | 16.8 | 13.6 |  | 21.4 | 23.2 | 21.3 | 17.7 | 20.9 |  |
| No | 78.4 | 69.4 | 77.2 | 82.2 | 86.0 |  | 74.9 | 70.6 | 75.7 | 80.0 | 78.9 |  |
| Missing | 2.1 | 4.9 | 1.9 | 1.0 | 0.4 |  |  |  |  |  |  |  |
| Smoking |  |  |  |  |  | <.0001 |  |  |  |  |  | <.0001 |
| never | 12.2 | 9.8 | 11.4 | 12.4 | 15.7 |  | 42.2 | 43.8 | 41.6 | 39.7 | 43.7 |  |
| former | 44.6 | 38.3 | 44.1 | 49.2 | 47.8 |  | 27.4 | 21.9 | 28.3 | 32.6 | 35.8 |  |
| current | 41.7 | 48.5 | 43.3 | 37.4 | 36.1 |  | 28.0 | 29.9 | 28.5 | 26.0 | 20.5 |  |
| missing | 1.5 | 3.4 | 1.2 | 1.0 | 0.4 |  | 2.4 | 4.4 | 1.7 | 1.8 | 0.0 |  |
| Physical activity |  |  |  |  |  | <.0001 |  |  |  |  |  | <.0001 |
| active | 43.8 | 34.4 | 42.2 | 48.1 | 51.9 |  | 41.8 | 29.5 | 45.2 | 51.6 | 54.7 |  |
| moderately active | 23.7 | 24.0 | 24.0 | 23.2 | 23.6 |  | 26.4 | 28.8 | 25.9 | 24.0 | 23.6 |  |
| little active | 22.9 | 29.0 | 23.7 | 19.9 | 17.9 |  | 21.3 | 26.5 | 20.0 | 16.1 | 16.5 |  |
| inactive | 9.6 | 12.4 | 10.0 | 8.8 | 6.7 |  | 10.0 | 14.4 | 8.5 | 8.0 | 5.1 |  |
| missing | 0.1 | 0.2 | 0.1 | 0.0 | 0.0 |  | 0.5 | 0.9 | 0.4 | 0.3 | 0.2 |  |
| Alcohol consumption | |  |  |  |  | <.0001 |  |  |  |  |  | <.0001 |
| abstainer | 13.6 | 22.0 | 13.8 | 11.0 | 6.9 |  | 33.8 | 50.0 | 29.6 | 20.7 | 16.5 |  |
| light drinker | 44.7 | 39.2 | 46.2 | 46.1 | 46.9 |  | 38.4 | 27.2 | 43.3 | 41.5 | 48.0 |  |
| moderate drinker | 23.8 | 16.2 | 21.6 | 26.6 | 32.5 |  | 12.8 | 7.9 | 12.7 | 20.0 | 22.2 |  |
| heavy drinker | 12.2 | 11.4 | 13.2 | 12.5 | 11.4 |  | 7.2 | 3.6 | 7.5 | 12.1 | 11.0 |  |
| missing | 5.7 | 11.1 | 5.3 | 3.8 | 2.3 |  | 7.8 | 11.3 | 6.9 | 5.7 | 2.4 |  |
| BMI |  |  |  |  |  | <.0001 |  |  |  |  |  | <.0001 |
| underweight (<20) | 2.7 | 3.1 | 2.6 | 2.7 | 2.4 |  | 6.0 | 4.2 | 6.0 | 6.8 | 11.4 |  |
| normal (20-25) | 47.7 | 41.7 | 42.9 | 50.3 | 58.5 |  | 50.0 | 40.5 | 51.9 | 58.0 | 63.0 |  |
| overweight (25.01-30) | 41.3 | 41.8 | 45.6 | 39.8 | 35.9 |  | 30.0 | 34.2 | 29.9 | 26.9 | 18.5 |  |
| obese (>30.01) | 4.7 | 5.9 | 5.7 | 4.9 | 1.9 |  | 8.9 | 12.6 | 7.9 | 5.7 | 5.1 |  |
| missing  *Psychosocial factors* | 3.6 | 7.4 | 3.2 | 2.4 | 1.4 |  | 5.2 | 8.4 | 4.3 | 2.6 | 2.0 |  |
|  |  |  |  |  |  |  |  |  |  |  |  |  |
| Negative life events |  |  |  |  |  | <.0001 |  |  |  |  |  | <.0001 |
| no negative life events | 51.8 | 46.1 | 53.6 | 51.0 | 55.6 |  | 51.2 | 49.4 | 53.2 | 51.3 | 46.3 |  |
| 1> negative life events | 28.8 | 29.1 | 27.9 | 31.3 | 27.6 |  | 27.9 | 27.2 | 27.3 | 28.8 | 33.3 |  |
| 2> negative life events | 17.4 | 20.1 | 16.8 | 16.7 | 16.3 |  | 17.1 | 17.3 | 16.2 | 18.0 | 19.9 |  |
| missing | 2.0 | 4.7 | 1.7 | 1.0 | 0.5 |  | 3.8 | 6.1 | 3.3 | 1.9 | 0.4 |  |
| Civil status |  |  |  |  |  | <.0001 |  |  |  |  |  | <.0001 |
| married | 83.8 | 76.4 | 85.7 | 87.0 | 85.6 |  | 74.5 | 68.9 | 78.9 | 72.9 | 71.3 |  |
| single | 5.7 | 9.8 | 4.6 | 3.6 | 4.8 |  | 5.3 | 4.3 | 3.6 | 9.2 | 14.0 |  |
| divorced | 6.1 | 7.2 | 5.7 | 5.5 | 6.0 |  | 7.7 | 7.1 | 7.4 | 9.0 | 9.6 |  |
| widowed | 2.9 | 3.4 | 2.9 | 2.6 | 2.5 |  | 11.1 | 18.1 | 8.8 | 7.4 | 4.5 |  |
| missing | 1.6 | 3.2 | 1.1 | 1.3 | 1.0 |  | 1.4 | 1.7 | 1.3 | 1.6 | 0.6 |  |
| Use of sleep/anxiety drugs |  |  |  |  |  | <.0001 |  |  |  |  |  | <.0001 |
| yes | 5.5 | 9.4 | 5.1 | 4.4 | 3.0 |  | 10.3 | 14.9 | 8.2 | 8.5 | 6.9 |  |
| no | 92.6 | 86.9 | 92.9 | 94.1 | 96.5 |  | 86.8 | 80.4 | 89.1 | 89.9 | 92.7 |  |
| missing | 2.0 | 3.7 | 2.1 | 1.5 | 0.5 |  | 3.0 | 4.7 | 2.6 | 1.6 | 0.4 |  |
| Depression, nervousness (vs. no) |  |  |  |  |  | <.0001 |  |  |  |  |  | <.0001 |
| yes | 12.5 | 13.5 | 11.9 | 12.9 | 11.8 |  | 14.6 | 16.0 | 13.2 | 14.5 | 18.1 |  |
| no | 85.2 | 82.0 | 85.7 | 85.6 | 87.5 |  | 82.1 | 78.4 | 84.3 | 84.0 | 80.5 |  |
| missing | 2.3 | 4.5 | 2.4 | 1.6 | 0.6 |  | 3.3 | 5.7 | 2.5 | 1.6 | 1.4 |  |
| SEP, socioeconomic position; BMI, body mass index (kg/m^2^)  ^a^ Adulthood socioeconomic position was determined by the respondent’s highest attained educational level, with 1= primary, 2= lower secondary, 3= higher secondary, 4=tertiary.  ^b^ P-values (determined by chi-square tests) show whether differences between the categories of a variable were significant. | | | | | | | | | | | | |
