# Supplementary material for: Socioeconomic inequalities in cardiovascular mortality and the role of childhood socioeconomic conditions and adulthood risk factors: a prospective cohort study with 17-years of follow up
Source: BMC Public Health. 2012 Dec 5;12:1045. doi: 10.1186/1471-2458-12-1045 (PMC3539932; doi:10.1186/1471-2458-12-1045)
Supplement: Additional file 2 — This Table shows the relative importance of the four health-behaviours to the explanation of adulthood SEP inequalities in CVD mortality, for men and women. [file 1471-2458-12-1045-S2.doc]

**Additional file 2**

**Supplementary Table B.** Role of smoking, physical activity, alcohol consumption, and BMI in explaining the associations of adulthood SEP**^a^** with CVD mortality (adjusted for age), among men and women

|  | **Adulthood SEP** | | | | | | |
| --- | --- | --- | --- | --- | --- | --- | --- |
| **Explanatory models**  ^b^ | **1- low** | %^c^ | **2- middle low** | %^c^ | **3- middle high** | %^c^ | **4 - high** |
|  | **HR (95% CI)** |  | **HR (95% CI)** |  | **HR (95% CI)** |  | **HR (95% CI)** |
| **Men** |  |  |  |  |  |  |  |
| Model 1 | 1.84  (1.41-2.39) |  | 1.32  (1.01-1.73) |  | 1.31  (0.97-1.76) |  | 1.00 |
|  |  |  |  |  |  |  |  |
| Model 3.1:  smoking | 1.70  (1.31-2.22) | -17% | 1.28  (0.98-1.68) | -13% | 1.31  (0.98-1.77) | - | 1.00 |
|  |  |  |  |  |  |  |  |
| Model 3.2:  physical activity | 1.81  (1.39-2.36) | -4% | 1.31  (1.00-1.72) | -3% | 1.32  (0.98-1.78) | - | 1.00 |
|  |  |  |  |  |  |  |  |
| Model 3:  smoking + physical activity | 1.70  (1.30-2.21) | - 17% | 1.28  (0.98-1.68) | -13% | 1.33  (0.99-1.79) | - | 1.00 |
| **Women** |  |  |  |  |  |  |  |
| Model 1 | 1.80  (1.04-3.10) |  | 1.39  (0.80-2.41) |  | 1.42  (0.77-2.62) |  | 1.00 |
|  |  |  |  |  |  |  |  |
| Model 3.1:  smoking | 1.66  (0.96-2.87) | -18% | 1.32  (0.76-2.29) | -18% | 1.37  (0.74-2.53) | -12% | 1.00 |
|  |  |  |  |  |  |  |  |
| Model 3.2:  physical activity | 1.60  (0.92-2.77) | -25% | 1.29  (0.74-2.23) | -26% | 1.36  (0.74-2.51) | -14% | 1.00 |
|  |  |  |  |  |  |  |  |
| Model 3.3:  alcohol consumption | 1.60  (0.92-2.78) | -25% | 1.32  (0.76-2.30) | -18% | 1.41  (0.76-2.60) | -2% | 1.00 |
|  |  |  |  |  |  |  |  |
| Model 3.4:  BMI | 1.76  (1.01-3.04) | -5% | 1.39  (0.80-2.41) | - | 1.44  (0.78-2.66) | - | 1.00 |
|  |  |  |  |  |  |  |  |
| Model 3:  smoking + physical activity + alcohol + BMI | 1.36  (0.78-2.38) | -55% | 1.20  (0.69-2.09) | -49% | 1.35  (0.73-2.50) | -17% | 1.00 |
| SEP, socioeconomic position; CVD, cardiovascular diseases; HR, hazard ratio; CI, confidence interval; BMI, body mass index (kg/m^2^)  ^a^ Adulthood socioeconomic position was determined by the respondent’s highest attained educational level, with 1= primary, 2= lower secondary, 3= higher secondary, 4=tertiary.  ^b^ Only behavioural factors that were significantly associated with CVD mortality and unequally distributed across adulthood SEP groups were included in the explanatory models. All models were adjusted for age.  ^c^ The percentages show the reduction in harzard ratio (HR) compared to model 1A, per SEP group. For instance, the reduction in the OR for the lowest adulthood SEP group among men when adding smoking to the model, is [(1.84-1.70)/(1.84-1.00)] * 100 = 17%. | | | | | | | |
